# Preparation protocols and periodontal applications of platelet rich fibrin, a review

**DOI:** 10.3389/fdmed.2026.1810175

**Published:** 2026-03-26

**Authors:** Sanabel O. Barakat, Nebras I. AlHawamdeh, Rama Marar

**Affiliations:** 1 Faculty of Dentistry, Zarqa University, Zarqa, Jordan; 2 Cairo University, Cairo, Egypt

**Keywords:** Alb-PRF, A-PRF, CTG substitues, i-PRF, Ly-PRF, PRF (platelet-rich fibrin), surgical periodontal therapy, T-PRF

## Abstract

Blood concentrates, such as platelet rich plasma and platelet rich fibrin, have been proposed to speed up tissue healing and regeneration in many dental fields. This was based on the fact that upon platelet activation, α-granules, which are reservoirs for many growth factors, fuse with the plasma membrane and release their cargo. The released growth factors exhibit chemotactic and mitogenic properties that promote and modulate cellular functions involved in tissue healing, cell proliferation, and tissue regeneration. Platelet rich fibrin (PRF) products were introduced as a grafting biomaterial to be used in surgical periodontics, because of their regenerative qualities. PRF variants are totally autogenous, easy to prepare, fast, economic and most importantly, they do not involve a donor site, therefore PRF was suggested as a connective tissue graft (CTG) substitute, to avoid complications relating to the second surgical site to harvest the CTG. It is important to highlight that the quality and the content of the PRF matrix depends largely on the preparation conditions of the patients’ blood sample. Based on the centrifugation speed of the extracted blood, different PRF matrices can be produced, such as Leukocyte- platelet rich fibrin (L-PRF), Advanced platelet rich fibrin (A-PRF) and Advanced platelet rich fibrin plus (A-PRF+), in addition to the injectable form of platelet rich fibrin (i-PRF) and lyophilised PRF (Ly-PRF). All these variants are currently used in different dental and periodontal procedures. This article aims to provide an overview of different PRF preparations and their applications in periodontal surgery.

## 1 Introduction

In periodontal surgery, connective tissue graft (CTG) is considered the gold standard soft tissue grafting material. Unfortunately, CTG procedures have inherent challenges, related to palatal donor site (1). For this, it has been advised to find a CTG substitute biomaterial that could do without a second operation. Currently, several biomaterials are available to overcome the shortcomings of the autologous CTG, including acellular dermal matrix, enamel matrix derivative, Platelet rich fibrin products (PRF), xenogenic dermal and collagen matrix (34).

PRF comprises a group of autologous platelet concentrates, and their biological properties are strongly dependent on preparation protocols.‏ Understanding the biological implications of different PRF preparations is essential for appropriate clinical application in periodontal surgery.

## 2 Platelet rich fibrin

PRF is an autologous growth factor reservoir that accumulates platelets, leukocytes, cytokines, and growth factors in a physiologic fibrin clot (2). Choukroun, introduced the first step towards the generation of autologous blood-derived PRF matrices that do not require anticoagulants. PRF can be formulated by the separation of blood after centrifugation. With the absence of anticoagulants, activation of platelets occurs due to contact with the tube wall and starts the coagulation cascade, where fibrinogen forms fibrin, in the presence of physiologic thrombin. PRF matrix can then be obtained at the middle of the tube, with massively concentrated platelets trapped within (3).

## 3 Significance of PRF

### 3.1 Role of fibrin clot

Fibrin plays a determining role in platelet aggregation during hemostasis as fibrin alone acts as a provisional matrix allowing cell invasion and tissue regeneration. This fibrin matrix leads directly to angiogenesis. PRF holds about 97% of platelets and 50% of leukocytes of the blood sample, in a three-dimensional distribution within the fibrin matrix (4). Since growth factors have an extremely short half-life span, the PRF fibrin matrix plays an important role in supporting slow and gradual release of these growth factors (active components) in the surgical site. In addition, the fibrin clot provides a scaffold for recruitment and migration of tissue cells to the site of injury.‏

The Fibrin clot provides a provisional matrix for influx of monocytes, fibroblasts and endothelial cells (5). Furthermore, naturally existing mesenchymal stem cells in the blood were found trapped in the fibrin clot of PRF. These cells have the potential to differentiate into other cell types and to express several growth factors including fibroblast growth factor (FGF) and vascular endothelial growth factor (VEGF). Hence, the ability of PRF to promote the proliferation of vascular endothelial cells, enhance vascular stability, and support a long-standing functional vascular network (6).

### 3.2 Role of platelets

Platelets are discoid, anucleated blood cells, derived from megakaryocytes and have an average lifespan of 8–12 days (7). Platelets serve a critical function in hemostasis at the site of disruptive vascular endothelium. These cells are replete with secretory granules, α-granules, dense granules and lysosomes that are essential for platelet function, as they provide a natural source of factors that help wound healing and tissue regeneration (43). Upon platelet activation, growth factors are released by α-granules which encompass a group of cytokine polypeptides with low molecular weight compounds including platelet-derived growth factor (PDGF), transforming growth factor-β, (TGF-β), Vascular endothelial growth factor (VEGF), Insulin-like growth factor-1 (IGF-1) and epidermal growth factor (EGF) (8).‏

### 3.3 Role of growth factors

PDGF is present in large amounts in platelet α-granules and is accumulated in high quantities in the PRF matrix. PDGF is an essential regulator for migration, proliferation and survival of mesenchymal cell lineages, also it promotes collagen production for remodeling of extracellular matrix during wound healing. PDGF has a mitogenic effect on stem cells and osteoblasts, it stimulates osteoprogenitor cells, replication of endothelial cells and promotes angiogenesis. Further, PDGF modulates the effect of other growth factors (8).

TGF-β is a superfamily of more than 30 members, with TGF-β1 and TGF-β2 the most significant isoforms. TGF-β mediates tissue repair, immune responses and extracellular matrix synthesis. Also, it is the most powerful fibrosis regulator amongst all cytokines, which makes it important for wound healing, angiogenesis, re-epithelization and connective tissue regeneration. TGF-β is crucial during bone formation contributing to osteoblast precursors chemotaxis and mitogenesis, and stimulates osteoblast activity) (8).

EGF stimulates chemotaxis of endothelial cells and angiogenesis, as well as mitosis of mesenchymal cells. It enhances epithelization and significantly shortens time needed for healing (2).

VEGF is secreted by activated thrombocytes. It promotes angiogenesis and stimulates new blood vessel formation. Thus, it increases blood flow and brings nutrients to the injured site) (8).

IGF-1 is a cell proliferative mediator since it acts as a positive regulator of proliferation and differentiation of most cell types: and it stimulates differentiation and mitogenesis of mesenchymal cells. IGF-I stimulates bone formation by increasing cellular bone matrix production (9). It is mention worthy that IGF-I is a cell-protective agent since it under-regulates programmed cell apoptosis, by inducing survival signals (44).

### 3.4 Role of leukocytes

Leukocytes trapped in the PRF membrane play an important role in wound healing. They have anti-infectious and immune regulation activities, via the secretion of key immune cytokines such as interleukins (IL) and tumor necrosis factor alpha (TNF-α) (7).‏

## 4 PRF preparations

### 4.1 Choukroun L-PRF

The initial protocol for PRF production, required 10 mL of venous blood collected from the patient without anticoagulants in a plain glass tube; and centrifuged at 2,700 revolutions per minute (rpm) at 708*g* relative centrifugation force (RCF) for 12 min. The produced fibrin network exhibits a dense structure with minimal inter-fibrous spaces (10).‏

### 4.2 A-PRF

To improve the quality of the PRF clot obtained, Ghanaati et al. (7) modified the preparation protocol by reducing the applied RCF and rpm. A-PRF was produced by spinning 10 mL blood sample at 1,500 rpm (RCF of 208*g*) for 14 min. In comparison with Choukroun's PRF mattress, histological and histo-morphometric study showed that the A-PRF has more porous structure with a larger interfibrous space compared to that of Choukroun's L-PRF, which enhances better cellular penetration into the fibrin clot and facilitates revascularization. Although platelets were detected throughout the clot in both types, more platelets were found in the distal part (away from the buffy coat), in A-PRF; unlike L-PRF, where the cells distribution pattern was mostly accentuated within the proximal part close to the buffy coat, and platelets density decreased towards the distal part. Reduction of RCF led not only to more even cellular distribution but also to an enhanced number of platelets and inflammatory cells. Neutrophils, T-lymphocytes, B-lymphocytes and monocytes were observed in significantly higher numbers in the A-PRF samples.‏

### 4.3 A-PRF+

A more improved variant of A-PRF, which was obtained by a slight reduction in the centrifugation speed and time, by spinning 10 mL of blood in glass tubes at 1,300 rpm (200*g* RCF) for 8 min. The “low speed centrifugation concept” (LSCC) in PRF matrix preparation showed a significant increase in growth factors content and release over 10 days in A-PRF+ compared to L_PRF. A-PRF+ showed significantly higher levels of human fibroblast migration and proliferation as well as higher levels of messenger ribonucleic acid (mRNA) for collagen type 1, Platelet derived growth factor (PDGF) and transforming growth factor (TGF); as compared to A-PRF and L-PRF (6). In terms of structural integrity of the clot, A-PRF+ showed similar porosity to A-PRF when the fibrin network was examined (11). According to Simos-Pedro et al. (12), Membranes prepared from the A-PRF+ with significantly increased maximal traction indicate a higher strength and viscoelasticity in periodontal and oral surgeries.

### 4.4 i-PRF

PRF variants are mostly dense matrices and cannot be injected except for the recently developed liquid i-PRF. i-PRF can be produced by spinning 10 mL of whole blood without anticoagulants at 700 rpm (60*g* RCF) for 3 min. For this purpose, plastic tubes are used, since plastic does not activate platelets like plain glass. Thus, the fluid form of PRF can be obtained without using anticoagulants (13).‏

### 4.5 Titanium platelet rich fibrin (T-PRF)

This is another form of PRF products described in the literature with proven efficacy in periodontal treatments. T-PRF is generated by the same protocol as L-PRF, except that sterilized titanium tubes are used for fibrin clot preparation, rather than plain glass tubes. When blood is centrifuged in titanium tubes, the resultant is a more densely woven, thicker, and firmer fibrin network that releases greater concentrations of growth factors such as VEGF, PDGF, and TGF. Thus, providing prolonged and stronger bioactivity, supporting angiogenesis, cell proliferation, and osteogenic differentiation (14).

### 4.6 Albumin PRF (Alb-PRF)

Very recently, a novel heating process was shown to extend the working properties of PRF from a standard 2 week period toward a duration of greater than 4 months (41). For this, peripheral blood is collected in 9-mL plastic tubes then centrifuged at 700*g* for 8 min. Thereafter, the platelet-poor plasma layer is heated at 75 °C for 10 min to create denatured albumin (albumin gel) (40). The remaining cells and growth factor found within the buffy coat layer (liquid PRF) are mixed back together with the cooled albumin gel to form Alb-PRF (15).

## 5 Factors affecting the quality of PRF

### 5.1 Preparation protocol

Studies that compared different PRF preparation protocols indicated that LSCC improves the clots structural porosity, enhances the platelet and leukocyte content and growth factors availability as well as their sustained release. Reducing centrifugation speed from 2,400 rpm (710*g*) to 1,200 rpm (177*g*) and finally to 600 rpm (44*g*) resulted in formulation of PRF matrices with highest amount of platelets, leukocytes and growth factors concentration (16).‏

### 5.2 Tube direction

To produce PRF membranes, the tube must be angulated at 45 degrees while spinning (10). Miron et al. (17), reported a significant increase in platelets and leukocytes numbers up to 3.5 times in horizontal L-PRF, A-PRF and i-PRF, as compared to angled centrifuges. Thereafter, horizontal platelet rich fibrin (H-PRF) was introduced as a concept.‏

### 5.3 Time

The size of the produced PRF clot is affected by the time elapsing between blood extraction and centrifugation. In one experiment, blood samples were centrifugated either immediately after collection or after 30, 60, 90, or 120 s. According to the results, a longer delay before centrifugation led to a smaller final size of PRF membranes. At 90 s following blood draw, a significant 13% reduction in PRF membrane size was observed and after 120 s a significant 23% reduction was reported. Therefore, blood collected from the patient must be immediately centrifuged to produce the possible largest PRF membrane (18).‏

### 5.4 Storage

PRF matrices cannot be stored because they are prepared without anticoagulants. Storage may shrink the PRF membranes and alter their structural integrity. Also, dehydration of PRF membranes results in reduction of the total content of growth factors as well as altering the biologic properties of leukocytes. To avoid bacterial contamination, PRF membranes cannot be refrigerated (19).‏

### 5.5 Lyophilization

Lyophilized-PRF (Ly-PRF) is a freeze-dried form of PRF designed to enhance storage ability and clinical availability. Ngah et al. (20) demonstrated that lyophilization preserves the fibrin membrane while producing a “spong-like” scafold with significantly larger pores than fresh PRF. thus, promotes cellular infiltration and tissue integration. Further, lyophilization extends PRF shelf life while maintaining its biological performance especially for bone regeneration and when delayed wound healing is anticipated.‏

### 5.6 Heat

Heating the liquid platelet-poor plasma layer from PRF tube affects the resorption properties of albumin (albumin gel) and can be extended from weeks to months (15).

### 5.7 Gender and age

It seems that patient's characteristics might influence the quality of PRF clot. According to Miron et al. (17), females have an average of 17% (*p* < 0.05) larger fibrin clot compared to males’ clots. Concerning age effect, although not statistically significant, the size of PRF membrane increased as the donor’s age increased.‏

### 5.8 Tube material

The quality of PRF matrix is significantly affected by the type of tube material, with additive-free plain glass tubes yielding high quality PRF membrane by activating Factor XII and initiating the natural coagulation process without chemical additives, this promotes a more natural distribution of the platelets within the PRF matrix (21). On the other hand, silica-coated plastic tubes have a negative impact on PRF products, as they can lead to smaller silica-contaminated clots. Also, Silica particles may cause cell apoptosis and reduced human cell proliferation. These cytotoxic particles contribute to a less desirable distribution of platelets (22). Regarding Silicone-coated tubes, they were related to inflammatory responses and delay in PRF clot formation process (23).‏ Titanium tube were also used for PRF preparation, According to Uzun et al. (14), platelets activated by titanium result in a more mature and aggregated form of PRF matrix, as the fibrin carpets had a firmer network structure and longer resorption time in the tissues compared to other PRF clots formed in glass tubes.‏

## 6 PRF and periodontal regeneration

This section presents selected examples illustrating the use of PRF in periodontology. It is not intended to provide a comprehensive review of all available applications, rather, it highlights clinical scenarios to demonstrate the potential scope of PRF uses.

The most common use of PRF matrices in periodontal procedures is for alveolar ridge preservation, PRF decreases post-operative pain and morbidity, accelerates hard and soft tissue healing particularly for implant placement, and lowers incidence of alveolar osteitis (15), It is advocated that in case of implant placement in fresh extraction sockets, to fill the jump gap with PRF membranes to maintain crestal bone height during osseointegration (24).

Regarding gingival recession treatment and coronally advanced flap (CAF), PRF may represent a valid treatment modality for exposed roots, given an adequate amount of keratinized tissue width (KTW) was available, compared to CAF alone (42). Culhaoglu et al. (25) evaluated the effect of the number of layers of PRF membranes on gingival recession treatment outcomes in comparison with CTG. Better root coverage was obtained when more PRF membranes were used with CAF. Interestingly, PRF results were comparable to CTG when multi-layered PRF (4 membranes) were used. In agreement, results of systematic review and meta-analysis showed that PRF significantly increased the percentage of root coverage and improved the clinical attachment level (CAL) compared to CAF alone, with no change on the KTW. Compared to PRF, CTG resulted in significantly better KTW and gingival recession reduction. It is worth mentioning that in this report no statistically significant differences were reported between PRF and enamel matrix derivatives or between PRF and amnion membrane, when used with CAF for root coverage (26).‏

PRF in periodontal soft tissue repair procedures favors soft tissue healing and periodontal ligament regeneration when compared to open flap debridement alone as it yields better results in terms of improved clinical attachment levels and reduced probing pocket depths, in treatment of furcation involvement (Type II) (27). In intra bony defects, PRF has a positive effect on CAL gain and increase in bone defect fill, determined either radiographically or by surgical re-entry (28). Actually, adding PRF to OFD debridement to treat intra-bony defect significantly reduces probing depth by about 1.27 mm (*p* < 0.00001) and improves clinical attachment level by about 1.24 mm (*p* < 0.00001), with significant increase in radiographic bone fill by about 1.57 mm (*p* < 0.00001) (26).

The effect of i-PRF on gingival phenotype modification was also tested. According to Idris et al. (29) four-time injections led to significant increase in keratinized tissue width after 6 months follow up. Nonetheless, the findings were clinically nonsignificant.

In the last few years, the effect of PRF membrane was clinically investigated for surgical reconstruction of receded interdental papilla (IDP) (30). In a recent randomized clinical trial, Barakat et al. (31), tested the effect of folded A- PRF membrane in the treatment of receded papillae. Their results showed that folded A-PRF membrane can be equally effective as CTG in increasing deficient IDP height with about 2 mm, and these results may be maintained for 1 year.

## 7 Discussion

In periodontal surgery, connective tissue graft (CTG) is considered the gold standard soft tissue grafting material (32), since it is suitable for soft tissue volume build-ups. CTG mechanically supports the periodontal flap and stabilizes it during wound healing. The presence of CTG under the flap reduces soft tissue contraction during the early phase of healing, which leads to a significantly greater tissue augmentation (1).‏

Unfortunately, CTG procedures have inherent challenges, with pain and post operative bleeding, related to palatal donor site, reported as the two most common complications. For this, it has been advised to find a CTG substitute biomaterial that could do without a second operation (33). Currently, several biomaterials, including PRF products, are available to overcome the shortcomings of CTG (34).

PRF and its variants are the second generation of blood concentrates, that has emerged in the last 3 decades, especially in regenerating soft tissue and bone lost due to periodontal disease (38). They are healing biomaterials with inherent regenerative capacity. PRF differs from its predecessor blood concentrates (PRP & PRGF) by the virtue of its composition, properties. single step preparation, and cost-effectiveness (39). PRF does not require the use of anticoagulants nor the activation by additional bovine thrombin making it fully autologous with fewer risks of immunologic reactions (35).‏

PRF membranes clinical benefits and biological potential are dependent upon the handling of extracted blood and preparation protocol. The centrifugation process plays an important role in determining the cellular contents as well as growth factors availability, which are crucial for tissue regeneration. Any variation in spin speed or tube material can affect clot quality in terms of fibrin density and configuration as well as growth factors content and release sustainability. It is now well established that with LSCC and reduction of the applied rcf, PRF quality improves leading to better clinical outcomes (36). Further, the concept of horizontal platelet rich fibrin (H-PRF) for the preparation of L-PRF, A-PRF and i-PRF, significantly increases in the number of platelets and as compared to angled centrifuges (17). Unless lyopfilized or extended, for best results, PRF matrices should be processed immediately after blood extraction and used directly into the patients’ mouth, as PRF products cannot be stored nor refrigerated.

Regarding uses of PRF products in periodontal procedures, recent reports showed that PRF matrices in conjunction with open flap debridement significantly improve clinical parameters compared to OFD alone. Combining PRF with bone grafting biomaterials enhances radiographic and clinical bone fill more than bone graft alone. PRF products are also used in treatment of gingival recession, IDP deficiency and furcation involvement, to overcome shortcomings of CTG based procedures (37). In general, PRF based procedures have shown promising results, especially if folded, PRF may be used as a CTG substitute in some clinical scenarios to achieve tissue regeneration with less morbidity (31).

However, lack of standardization of PRF preparation across clinical studies and the inconsistencies in some reported results, highlights the need to a unified protocol to ensure reproducibility of high quality of PRF membranes and predictability of clinical benefits.

## 8 Future perspectives

Despite the available evidence supporting using PRF in periodontal therapy, there are several gaps in knowledge that require future research focusing on:

- Patient-centered outcomes such as pain, esthetics and cost.
- Standardizing PRF preparation protocols for different periodontal treatments.

## References

[1] Zucchelli G Tavelli L McGuire MK Rasperini G Feinberg SE Wang HL Autogenous soft tissue grafting for periodontal and peri-implant plastic surgical reconstruction. J Periodontol. (2020) 91(1):9–16. 10.1002/JPER.19-0350 31461778

[2] Salama M Ashour M Taher E Rashed F Ibrahim M El-Nabalawy M Effect of auto logous platelet rich plasma on the fertility and quakity of cryopreserved Buffalo bull semen: a comparative study using OptiXcell and tris egg yalk extenders. BMC Vet Res. (2024) 20:250. 10.1186/s12917-024-04022-x 38849855 PMC11157829

[3] Choukroun J Adda F Schoeffeler C Vervelle A. Opportunity for PRF in implant dentistry. J Implantodontie. (2001) 42:55–62.

[4] Dohan Ehrenfest DM del Corso M Diss A Mouhyi J Charrier J-B. Three-dimensional architecture and cell composition of a Choukroun’s platelet-rich fibrin clot and membrane. J Periodontol. (2010) 81(4):546–55. 10.1902/jop.2009.090531 20373539

[5] Clark RAF. Fibrin and wound healing. Ann N Y Acad Sci. (2001) 936:355–67. 10.1111/j.1749-6632.2001.tb03522.x 11460492

[6] Kobayashi E Flückiger L Fujioka-Kobayashi M Sawada K Sculean A Schaller B Comparative release of growth factors from PRP, PRF, and advanced-PRF. Clin Oral Investig. (2016) 20(9):2353–60. 10.1007/s00784-016-1719-1 26809431

[7] Ghanaati S Booms P Orlowska A Kubesch A Lorenz J Rutkowski J Advanced platelet-rich fibrin: a new concept for cell- based tissue engineering by means of inflammatory cells. J Oral Implantol. (2014) 40(6):679–89. 10.1563/aaid-joi-D-14-00138 24945603

[8] Pavlovic V Ciric M Jovanovic V Trandafilovic M Stojanovic P. Platelet-rich fibrin: basics of biological actions and protocol modifications. Open Med. (2021) 16(1):446–54. 10.1515/med-2021-0259 PMC7985567 33778163

[9] Rattanawonsakul K Seleiro G Workman V Claeyssens F Bolt R Seemaung P The effect of liquid platelet-rich fibrin on oral cells and tissue-engineered oral mucosa models in vitro. Sci Rep. (2025) 15:26076. 10.1038/s41598-025-11868-0 40681824 PMC12274383

[10] Dohan DM Choukroun J Diss A Dohan SL Dohan AJJ Mouhyi J Platelet-rich fibrin (PRF): a second-generation platelet concentrate. Part I: technological concepts and evolution. Oral Surg Oral Med Oral Pathol Oral Radiol Endod. (2006) 101(3):37–44. 10.1016/j.tripleo.2005.07.008 16504849

[11] El Bagdadi K Kubesch A Yu X Al-Maawi S Orlowska A Dias A Reduction of relative centrifugal forces increases growth factor release within solid platelet-rich-fibrin (PRF)-based matrices: a proof of concept of LSCC (low speed centrifugation concept). Eur J Trauma Emerg Surg. (2019) 45(3):467–79. 10.1007/s00068-017-0785-7 28324162 PMC6579868

[12] Simões-Pedro M Tróia PMBPS Dos Santos NBM Completo AMG Castilho RM de Oliveira Fernandes GV. Tensile strength essay comparing three different platelet-rich fibrin membranes (L-PRF, A-PRF, and A-PRF+): a mechanical and structural in vitro evaluation. Polymers. (2022) 14(7):1392. 10.3390/polym14071392 35406263 PMC9002533

[13] Abd El Raouf M Wang X Miusi S Chai J Mohamed AbdEl-Aal AB Nefissa Helmy MM Injectable-platelet rich fibrin using the low speed centrifugation concept improves cartilage regeneration when compared to platelet-rich plasma. Platelets. (2019) 30(2):213–21. 10.1080/09537104.2017.1401058 29240523

[14] Uzun BC Ercan E Tunalı M. Effectiveness and predictability of titanium-prepared platelet-rich fibrin for the management of multiple gingival recessions. Clin Oral Investig. (2018) 22(3):1345–54. 10.1007/s00784-017-2211-2 28990126

[15] Kobayashi M Miron R Moraschini V Zhang Y Gruber R Wang H. Efficacy of platelet-rich fibrin on socket healing after mandibular third molar extractions. J Oral Maxillofac Surg Med Pathol. (2021) 33(4):379–88. 10.1016/j.ajoms.2021.01.006

[16] Choukroun J Ghanaati S. Reduction of relative centrifugation force within injectable platelet-rich-fibrin (PRF) concentrates advances patients’ own inflammatory cells, platelets and growth factors: the first introduction to the low speed centrifugation concept. Eur J Trauma Emerg Surg. (2018) 44(1):87–95. 10.1007/s00068-017-0767-9 28283682 PMC5808086

[17] Miron RJ Chai J Zheng S Feng M Sculean A Zhang Y. A novel method for evaluating and quantifying cell types in platelet rich fibrin and an introduction to horizontal centrifugation. J Biomed Mater Res A. (2019) 107(10):2257–71. 10.1002/jbm.a.36734 31148358

[18] Miron RJ Dham A Dham U Zhang Y Pikos MA Sculean A. The effect of age, gender, and time between blood draw and start of centrifugation on the size outcomes of platelet-rich fibrin (PRF) membranes. Clin Oral Investig. (2019) 23(5):2179–85. 10.1007/s00784-018-2673-x 30280327

[19] Sahu K Jadhav S Nabi Khan SS Singh N Khan M Agarwal A. Choukroun’s platelet-rich fibrin (L-PRF): a benevolence to surgical and reconstructive dentistry. Int J Oral Care Res. (2020) 8(3):45. 10.4103/INJO.INJO_26_20

[20] Ngah NA Dias GJ Tong DC Mohd Noor SNF Ratnayake J Cooper PR Lyophilised platelet-rich fibrin: physical and biological characterisation. Molecules. (2021) 26:7131. 10.3390/molecules26237 34885714 PMC8658988

[21] Miron R Kawase T Dham A Zhang Y Kobayashi M Sculean A. A technical note on contamination from PRF tubes containing silica and silicone. BMC Oral Health. (2021) 21(1):135. 10.1186/s12903-021-01497-0 33740959 PMC7980632

[22] Bains V Mahendra J Mittal M Bedi M Mahendra L. Technical considerations in obtaining platelet rich fibrin for clinical and periodontal research. J Oral Biol Craniofac Res. (2023) 13(6):714–9. 10.1016/j.jobcr.2023.09.003 37731846 PMC10507643

[23] Liu M Liu Y Luo F. The role and mechanism of platelet rich fibrin in alveolar bone regeneration. Biomed Pharmacother. (2023) 168:115795. 10.1016/j.biopha.2023.115795 37918253

[24] Avula KK Rayavaram UD Gujjula S Dandu SSPR Egatela P Nagireddy RR. The effect of platelet-rich fibrin on soft tissue and crestal bone in one-stage implant placement in fresh extraction sockets: a randomized controlled clinical trial. J Orofac Sci. (2018) 10(2):112–6. 10.4103/jofs.jofs_132_18

[25] Culhaoglu R Taner L Guler B. Evaluation of the effect of dose dependent platelet rich fibrin membrane on treatmne tof gingival recession: a randomized controlled clinical trial. J Appl Oral Sci. (2018) 26. 10.1590/1678-7757-2017-0278 29768524 PMC5958936

[26] Miron R Moraschini V Estrin N Shibli J Cosgarea R Jepsen K Autogenous platelet concentrates for treatment of intrabony defects-a systematic review with meta-analysis. Periodontol 2000. (2025) 97:153–90. 10.1111/prd.12598 39425513 PMC11808470

[27] Miron RJ Zucchelli G Pikos MA Salama M Lee S Guillemette V Use of platelet-rich fibrin in regenerative dentistry: a systematic review. Clin Oral Investig. (2017) 21(6):1913–27. 10.1007/s00784-017-2133-z 28551729

[28] Madi M Elakel AM. The clinical implications of platelet-rich fibrin on periodontal regeneration: a systematic review. Saudi Dent J. (2021) 33(2):55–62. 10.1016/j.sdentj.2020.12.002 33551617 PMC7848804

[29] Idris M Burhan A Hajeer M Sultan K Nawaya F. Efficacy of the injectable platelet-rich fibrin (i-PRF) in gingival phenotype modification: a systematic review and meta-analysis of randomized controlled trials. BMC Oral Health. (2024) 24:1331. 10.1186/s12903-024-05109-5 39487452 PMC11529032

[30] Barakat S. Interdental papilla recession and reconstruction of the missing triangle, review of the current literature. Front Dent Med. (2025) 5:1537452. 10.3389/fdmed.2024.1537452 39917646 PMC11797962

[31] Barakat SO Tawfik OK El-Kholy S ElNahass H. Evaluation of advanced platelet rich fibrin compared to connective tissue graft in the surgical management of papilla recession: a randomized controlled trial. Clin Oral Investig. (2024) 28(1):87–97. 10.1007/s00784-023-05486-1 38206354

[32] Chambrone L Botelho J Machado V Mascarenhas P Mendes JJ Avila-Ortiz G. Does the subepithelial connective tissue graft in conjunction with a coronally advanced flap remain as the gold standard therapy for the treatment of single gingival recession defects? A systematic review and network meta-analysis. J Periodontol. (2022) 93(9):1336–52. 10.1002/JPER.22-0167 35451068

[33] Vallecillo C Toledano-Osorio M Vallecillo-Rivas M Toledano M Rodriguez-Archilla A Osorio R. Collagen matrix vs. autogenous connective tissue graft for soft tissue augmentation: a systematic review and meta-analysis. Polymers. (2021) 13(11):1810. 10.3390/polym13111810 34072698 PMC8199411

[34] Moraschini V Calasans-Maia MD Dias AT De Carvalho Formiga M Sartoretto SC Sculean A Effectiveness of connective tissue graft substitutes for the treatment of gingival recessions compared with coronally advanced flap: a network meta-analysis. Clin Oral Invest. (2020) 24(10):3395–406. 10.1007/s00784-020-03547-3 32851531

[35] Niemczyk W Janik K Zurek J Skaba D Wiench R. Platelet rich plasma (PRP) and injectable platelet rich fibrin (i-PRF) in the non-surgical treatment of periodontitis – a systematic review. Int J Mol Sci (2024) 25:6319. 10.3390/ijms25126319 38928026 PMC11203877

[36] Miron R Xu H Chai J Wang J Zheng S Feng M Comparison of platelet rich fibrin (PRF) produced using 3 commertially available centrifuges at both high (∼700 g) and low (∼200 g) relative centrifugation forces. Clin Oral Investig. (2019) 24(3):1171–82. 10.1007/s00784-019-02981-2 31321574

[37] Miron RJ Moraschini V del Fabbro M Piattelli A Fujioka-Kobayashi M Zhang Y Use of platelet-rich fibrin for the treatment of gingival recessions: a systematic review and meta-analysis. Clin Oral Investig. (2020) 24(8):2543–57. 10.1007/s00784-020-03400-7 32591868

[38] Agrawal AA. Evolution, current status and advances in application of platelet concentrate in periodontics and implantology. World J Clin Cases. (2017) 5(5):159. 10.12998/wjcc.v5.i5.159 28560233 PMC5434315

[39] Bansal S Garg A Khurana R Chhabra P. Platelet-rich fibrin or platelet-rich plasma – which one is better? an opinion. Indian J Dent Sci. (2017) 9(5):49. 10.4103/IJDS.IJDS_55_17

[40] Fujioka-Kobayashi M Schaller B Mourão CFAB Zhang Y Sculean A Miron RJ. Biological characterization of an injectable platelet-rich fibrin mixture consisting of autologous albumin gel and liquid platelet-rich fibrin (alb-PRF). Platelets. (2021) 32(1):74–81. 10.1080/09537104.2020.1717455 31959025

[41] Miron RJ Pikos MA Estrin NE Kobayashi-Fujioka M Espinoza AR Basma H Extended platelet-rich fibrin. Periodontol 2000. (2024) 94(1):114–30. 10.1111/prd.12537 37986559

[42] Saleh W Abdelhaleem M Elmeadawy S. Assessing the effectiveness of advanced platelet rich fibrin in treating gingival recession: a systematic review and meta-analysis. BMC Oral Health. (2024) 24(1):1400. 10.1186/s12903-024-05115-7 39563291 PMC11575048

[43] Goel A Windsor L Gregory R Blanchard S Hamada Y. Effects of platelet rich fibrin on human gingival and periodontal ligament fibroblasts proliferation frm chronic periodontitis versus periodontally healthy subjects. ClinExpDentRes. (2021) 7:436–42. 10.1002/cre2.370 PMC8404503 33443821

[44] Crisci A. The L-PRF membrane (fibrin rich in platelets and leukocytes) and its derivatives (A-PRF, I-PRF) are useful as a source of stem cells in regenerative wound therapy: experimental work on the horse. Regen. Med. (2019) 3(1).

